# Bis[4-hy­droxy-3,5-dimeth­oxy­benzalde­hyde (2,4-dinitro­phen­yl)hydrazone] *N*,*N*-dimethyl­formamide disolvate monohydrate

**DOI:** 10.1107/S1600536810037803

**Published:** 2010-09-30

**Authors:** Lin-xiu Zhao, Gang-shen Li

**Affiliations:** a College of Chemical Engineering and Environment, North University of China, Taiyuan 030051, People’s Republic of China; b Key Laboratory of Surface and Interface Science of Henan, School of Material & Chemical Engineering, Zhengzhou University of Light Industry, Zhengzhou 450002, People’s Republic of China

## Abstract

In the title compound, 2C_15_H_14_N_4_O_7_·2C_3_H_7_NO·H_2_O, the hydrazone mol­ecules are roughly planar, with the two benzene rings twisted slightly relative to each other by dihedral angle of 6.04 (11) and 7.75 (11)° in the two mol­ecules. The water mol­ecule is linked to the Schiff base mol­ecule by an O—H⋯O hydrogen bond. Intra­molecular N—H⋯O hydrogen bonds occur. In the crystal, mol­ecules are linked by inter­molecular N—H⋯O and O—H⋯O hydrogen bonds.

## Related literature

For general properties of phenyl­hydrazone derivatives, see: Okabe *et al.* (1993 ▶). For related structures, see: Ohba (1996 ▶); Baughman *et al.* (2004 ▶); Kuleshova *et al.* (2003 ▶); Szczesna & Urbanczyk-Lipkowska (2002 ▶); Zhen & Han (2005 ▶).
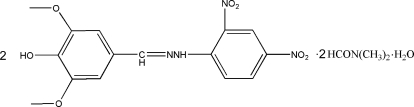


## Experimental

### 

#### Crystal data

2C_15_H_14_N_4_O_7_·2C_3_H_7_NO·H_2_O
*M* *_r_* = 888.81
Triclinic, 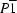

*a* = 12.208 (2) Å
*b* = 13.931 (2) Å
*c* = 14.537 (2) Å
α = 62.405 (16)°
β = 74.412 (15)°
γ = 72.171 (16)°
*V* = 2062.1 (6) Å^3^
*Z* = 2
Mo *K*α radiation
μ = 0.12 mm^−1^
*T* = 293 K
0.20 × 0.18 × 0.17 mm

#### Data collection

Bruker SMART CCD area detector diffractometer
Absorption correction: multi-scan (*SADABS*; Bruker, 1998 ▶) *T*_min_ = 0.975, *T*_max_ = 0.978
15349 measured reflections
8436 independent reflections
3010 reflections with *I* > 2σ(*I*)
*R*_int_ = 0.028

#### Refinement

*R*[*F* ^2^ > 2σ(*F* ^2^)] = 0.035
*wR*(*F* ^2^) = 0.071
*S* = 0.65
8417 reflections
578 parameters
H-atom parameters constrained
Δρ_max_ = 0.14 e Å^−3^
Δρ_min_ = −0.15 e Å^−3^

### 

Data collection: *SMART* (Bruker, 1998 ▶); cell refinement: *SAINT* (Bruker, 1998 ▶); data reduction: *SAINT*; program(s) used to solve structure: *SHELXTL* (Sheldrick, 2008 ▶); program(s) used to refine structure: *SHELXL97* (Sheldrick, 2008 ▶); molecular graphics: *ORTEPIII* (Burnett & Johnson, 1996 ▶); *ORTEP-3 for Windows* (Farrugia, 1997 ▶) and *XP* in *SHELXTL*; software used to prepare material for publication: *SHELXL97*.

## Supplementary Material

Crystal structure: contains datablocks global, I. DOI: 10.1107/S1600536810037803/dn2603sup1.cif

Structure factors: contains datablocks I. DOI: 10.1107/S1600536810037803/dn2603Isup2.hkl

Additional supplementary materials:  crystallographic information; 3D view; checkCIF report

## Figures and Tables

**Table 1:** Hydrogen-bond geometry (Å, °)

*D*—H⋯*A*	*D*—H	H⋯*A*	*D*⋯*A*	*D*—H⋯*A*
O2—H2⋯O16	0.82	1.89	2.610 (2)	145
N2—H2*A*⋯O4	0.86	2.05	2.637 (2)	125
N2—H2*A*⋯O17^i^	0.86	2.40	3.1141 (19)	140
O10—H10⋯O8	0.82	1.89	2.604 (2)	145
O10—H10⋯O11	0.82	2.27	2.7030 (17)	113
N7—H7⋯O12	0.86	2.06	2.642 (2)	125
N7—H7⋯O17	0.86	2.39	3.118 (2)	143
O17—H1*A*⋯O9^ii^	0.84	2.22	2.9936 (16)	153
O17—H1*A*⋯O10^ii^	0.84	2.42	3.1042 (17)	139
O17—H1*C*⋯O1	0.84	2.34	3.0790 (19)	147
O17—H1*C*⋯O2	0.84	2.44	3.1811 (18)	147

Symmetry codes: (i) 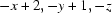; (ii) 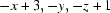.

**Fig. 1.**
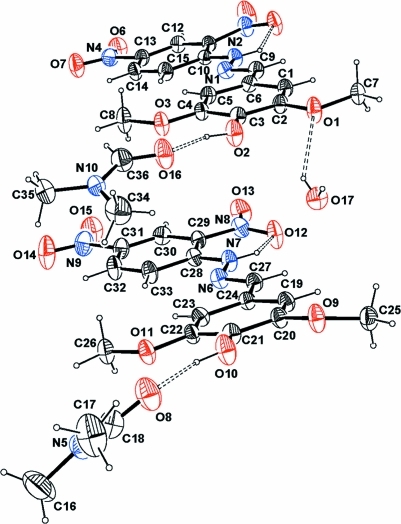
The asymmetric unit of (I) with the atom-labelling scheme. Displacement ellipsoids are drawn at the 30% probability level. H atoms are represented as small spheres of arbitrary radii. Hydrogen bonds are shown as dashed lines.

**Fig. 2.**
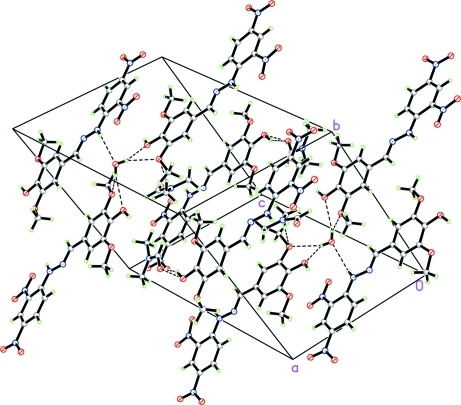
The molecular packing of the title compound,Hydrogen bonds are shown as dashed lines. H atoms not involved in hydrogen bondings have been omitted for clarity.

## supplementary crystallographic information

### Comment

2,4-Dinitrophenylhydrazine is a reagent which is widely used for condensation with aldehydes and ketones. Several phenylhydrazone derivatives have been shown to be potentially DNA-damaging and are mutagenic agents (Okabe *et al.* 1993). Structural information for phenylhydrazone derivatives is useful in studying their coordination properties. As part of our work, we have synthesized the title compound and report the crystal structure.

The asymmetric unit of the title compound, C_36_H_44_N_10_O_17_, is built up from two independent but roughly identical 4-hydroxy-3,5-dimethoxybenzaldehyde 2,4-dinitrophenylhydrazone, two dimethylformamide and one water molecules (Fig. 1). The hydrazone molecules are almost planar with the two benzene rings slightly twisted to each other by a dihedral angle of 6.04 (11)° and 7.75 (11)° respectivey. Bond lengths and bond angles agree with those of other dinitrophenylhydrazone derivatives(Ohba,1996; Baughman *et al.*, 2004; Kuleshova *et al.*, 2003; Szczesna & Urbanczyk-Lipkowska, 2002; Zhen & Han, 2005).

The Schiff base and the DMF molecules are linked through intermolecular O—H···O hydrogen bonds. There are intramolecular N—H···O hydrogen bond within the hydrazone molecules. Further O-H···O involving the water molecules help to stabilize the packing (Table 1, Fig. 2).

### Experimental

2,4-dinitrophenylhydrazine (1 mmol, 0.198 g) was dissolved in anhydrous ethanol (10 ml), H_2_SO_4_(98%, 0.5 ml) was then added and The mixture was stirred for several minitutes at 351k, 4-hydroxy-3,5-dimethoxybenzaldehyde (1 mmol, 0.182 g) in ethanol (5 mm l) was added dropwise and the mixture was stirred at refluxing temperature for 2 h. The product was isolated and recrystallized from DMF and water, red single crystals of (I) was obtained after three weeks.

### Refinement

All H atoms attached to C, N and O(hydroxyl) atoms were fixed geometrically and treatedas riding with C—H = 0.96 Å (methyl) or 0.93 Å (aromatic) and N—H =0.86 Å with U_iso_(H) = 1.2U_eq_(Caromatic or N) or U_iso_(H) = 1.5U_eq_(Cmethyl, O). H atoms of water molecule were located in difference Fourier maps and included in the subsequent refinement using restraints (O-H= 0.85 (1)Å and H···H= 1.39 (2)Å) with U_iso_(H) = 1.5U_eq_(O). In the last cycles of refinement, they were treated as riding on their parent O atom.

### Crystal data

**Table:**

2C_15_H_14_N_4_O_7_·2C_3_H_7_NO·H_2_O	*Z* = 2
*M**_r_* = 888.81	*F*(000) = 932
Triclinic, *P*1	*D*_x_ = 1.431 Mg m^−^^3^
Hall symbol: -P 1	Mo *K*α radiation, λ = 0.71073 Å
*a* = 12.208 (2) Å	Cell parameters from 3010 reflections
*b* = 13.931 (2) Å	θ = 3.3–26.3°
*c* = 14.537 (2) Å	µ = 0.12 mm^−^^1^
α = 62.405 (16)°	*T* = 293 K
β = 74.412 (15)°	Block, red
γ = 72.171 (16)°	0.20 × 0.18 × 0.17 mm
*V* = 2062.1 (6) Å^3^	

### Data collection

**Table:**

Bruker SMART CCD area detector diffractometer	8436 independent reflections
Radiation source: fine-focus sealed tube	3010 reflections with *I* > 2σ(*I*)
graphite	*R*_int_ = 0.028
ω scans	θ_max_ = 26.4°, θ_min_ = 3.1°
Absorption correction: multi-scan *SADABS*(Bruker, 1998)	*h* = −15→15
*T*_min_ = 0.975, *T*_max_ = 0.978	*k* = −17→17
15349 measured reflections	*l* = −18→18

### Refinement

**Table:**

Refinement on *F*^2^	Primary atom site location: structure-invariant direct methods
Least-squares matrix: full	Secondary atom site location: difference Fourier map
*R*[*F*^2^ > 2σ(*F*^2^)] = 0.035	Hydrogen site location: inferred from neighbouring sites
*wR*(*F*^2^) = 0.071	H-atom parameters constrained
*S* = 0.65	*w* = 1/[σ^2^(*F*_o_^2^) + (0.0322*P*)^2^] where *P* = (*F*_o_^2^ + 2*F*_c_^2^)/3
8417 reflections	(Δ/σ)_max_ = 0.001
578 parameters	Δρ_max_ = 0.14 e Å^−^^3^
0 restraints	Δρ_min_ = −0.15 e Å^−^^3^

### Special details

**Table:**

Geometry. All esds (except the esd in the dihedral angle between two l.s. planes) are estimated using the full covariance matrix. The cell esds are taken into account individually in the estimation of esds in distances, angles and torsion angles; correlations between esds in cell parameters are only used when they are defined by crystal symmetry. An approximate (isotropic) treatment of cell esds is used for estimating esds involving l.s. planes.
Refinement. Refinement of *F*^2^ against ALL reflections. The weighted *R*-factor *wR* and goodness of fit *S* are based on *F*^2^, conventional *R*-factors *R* are based on *F*, with *F* set to zero for negative *F*^2^. The threshold expression of *F*^2^ > σ(*F*^2^) is used only for calculating *R*-factors(gt) *etc*. and is not relevant to the choice of reflections for refinement. *R*-factors based on *F*^2^ are statistically about twice as large as those based on *F*, and *R*- factors based on ALL data will be even larger.

### Fractional atomic coordinates and isotropic or equivalent isotropic displacement parameters (Å^2^)

**Table:**

	*x*	*y*	*z*	*U*_iso_*/*U*_eq_	
O1	1.16438 (10)	0.47386 (11)	0.11183 (10)	0.0624 (4)	
O2	1.07317 (10)	0.42369 (12)	0.30577 (10)	0.0659 (4)	
H2	1.0419	0.4212	0.3641	0.099*	
O3	0.85512 (10)	0.51899 (11)	0.36572 (10)	0.0569 (4)	
O4	0.60331 (12)	0.98455 (12)	−0.26044 (10)	0.0728 (4)	
O5	0.43491 (13)	1.08306 (14)	−0.29372 (12)	0.0977 (6)	
O6	0.10800 (11)	1.07985 (13)	−0.03146 (13)	0.0778 (5)	
O7	0.13071 (12)	0.99503 (14)	0.13233 (14)	0.0843 (5)	
N1	0.69782 (12)	0.77314 (13)	0.01660 (11)	0.0448 (4)	
N2	0.63799 (12)	0.84473 (12)	−0.06680 (12)	0.0453 (4)	
H2A	0.6722	0.8612	−0.1305	0.054*	
N3	0.49985 (15)	1.01377 (15)	−0.23198 (14)	0.0577 (5)	
N4	0.16778 (14)	1.01869 (15)	0.03847 (17)	0.0588 (5)	
C1	0.98729 (14)	0.60679 (15)	0.04999 (15)	0.0424 (5)	
H1B	1.0192	0.6253	−0.0203	0.051*	
C2	1.05264 (15)	0.52930 (16)	0.12764 (16)	0.0433 (5)	
C3	1.00473 (15)	0.50120 (16)	0.23327 (15)	0.0430 (5)	
C4	0.89093 (15)	0.55258 (16)	0.25985 (16)	0.0409 (5)	
C5	0.82687 (14)	0.62989 (15)	0.18204 (15)	0.0437 (5)	
H5A	0.7513	0.6645	0.1997	0.052*	
C6	0.87395 (14)	0.65703 (15)	0.07682 (16)	0.0389 (5)	
C7	1.22460 (14)	0.50572 (16)	0.00683 (14)	0.0530 (6)	
H7B	1.3025	0.4619	0.0071	0.079*	
H7C	1.2270	0.5827	−0.0228	0.079*	
H7D	1.1847	0.4940	−0.0345	0.079*	
C8	0.73980 (15)	0.56672 (18)	0.39943 (15)	0.0712 (7)	
H8A	0.7263	0.5385	0.4746	0.107*	
H8B	0.6853	0.5480	0.3768	0.107*	
H8C	0.7300	0.6458	0.3696	0.107*	
C9	0.80494 (15)	0.73386 (15)	−0.00621 (15)	0.0446 (5)	
H9A	0.8391	0.7543	−0.0761	0.053*	
C10	0.52455 (15)	0.88791 (15)	−0.04531 (15)	0.0381 (5)	
C11	0.45313 (15)	0.96713 (16)	−0.12119 (15)	0.0410 (5)	
C12	0.33727 (15)	1.00841 (16)	−0.09368 (16)	0.0455 (5)	
H12A	0.2922	1.0600	−0.1455	0.055*	
C13	0.28912 (15)	0.97383 (17)	0.00874 (17)	0.0455 (5)	
C14	0.35523 (16)	0.89655 (16)	0.08688 (15)	0.0516 (6)	
H14A	0.3216	0.8734	0.1571	0.062*	
C15	0.46907 (16)	0.85492 (16)	0.06046 (15)	0.0497 (5)	
H15A	0.5122	0.8029	0.1136	0.060*	
O9	1.60110 (10)	−0.20303 (11)	0.61612 (10)	0.0575 (4)	
O10	1.50993 (10)	−0.25838 (13)	0.81097 (10)	0.0650 (4)	
H10	1.4725	−0.2779	0.8702	0.098*	
O11	1.27984 (10)	−0.19778 (11)	0.87289 (10)	0.0530 (4)	
O12	1.00060 (11)	0.22634 (13)	0.25371 (10)	0.0710 (5)	
O13	0.83852 (12)	0.34266 (12)	0.22412 (10)	0.0715 (4)	
O14	0.56221 (14)	0.28231 (17)	0.65779 (15)	0.1112 (7)	
O15	0.53231 (12)	0.36640 (14)	0.49623 (14)	0.0901 (6)	
N6	1.11811 (12)	0.05058 (12)	0.52678 (11)	0.0437 (4)	
N7	1.05646 (11)	0.12124 (12)	0.44502 (11)	0.0459 (4)	
H7	1.0884	0.1369	0.3808	0.055*	
N8	0.90520 (15)	0.27239 (15)	0.28452 (13)	0.0508 (5)	
N9	0.59430 (15)	0.30440 (18)	0.56430 (19)	0.0734 (6)	
C19	1.41392 (14)	−0.10112 (15)	0.55671 (14)	0.0421 (5)	
H19A	1.4459	−0.0826	0.4865	0.051*	
C20	1.48356 (15)	−0.16602 (16)	0.63432 (15)	0.0415 (5)	
C21	1.43604 (15)	−0.19613 (16)	0.73928 (15)	0.0423 (5)	
C22	1.31679 (15)	−0.16104 (15)	0.76710 (15)	0.0408 (5)	
C23	1.24810 (14)	−0.09458 (15)	0.69001 (15)	0.0402 (5)	
H23A	1.1690	−0.0698	0.7083	0.048*	
C24	1.29642 (14)	−0.06399 (15)	0.58421 (15)	0.0368 (5)	
C25	1.65080 (14)	−0.20819 (16)	0.51718 (15)	0.0559 (6)	
H25A	1.7314	−0.2446	0.5181	0.084*	
H25B	1.6441	−0.1344	0.4625	0.084*	
H25C	1.6102	−0.2490	0.5045	0.084*	
C26	1.15956 (15)	−0.16295 (17)	0.90709 (15)	0.0584 (6)	
H26A	1.1445	−0.1954	0.9824	0.088*	
H26B	1.1133	−0.1864	0.8805	0.088*	
H26C	1.1399	−0.0836	0.8814	0.088*	
C27	1.22455 (15)	0.00984 (15)	0.50237 (14)	0.0420 (5)	
H27A	1.2563	0.0273	0.4322	0.050*	
C28	0.94411 (15)	0.16467 (16)	0.46994 (15)	0.0390 (5)	
C29	0.86909 (15)	0.24109 (16)	0.39592 (15)	0.0390 (5)	
C30	0.75571 (15)	0.28574 (16)	0.42727 (16)	0.0474 (5)	
H30A	0.7081	0.3364	0.3772	0.057*	
C31	0.71345 (15)	0.25594 (18)	0.53100 (18)	0.0513 (6)	
C32	0.78217 (17)	0.17783 (18)	0.60649 (16)	0.0591 (6)	
H32A	0.7516	0.1556	0.6774	0.071*	
C33	0.89359 (15)	0.13397 (17)	0.57704 (15)	0.0508 (6)	
H33A	0.9387	0.0820	0.6287	0.061*	
O8	1.48326 (14)	−0.35737 (15)	1.01517 (13)	0.0855 (5)	
N5	1.44236 (15)	−0.46103 (15)	1.18764 (15)	0.0591 (5)	
C16	1.3621 (2)	−0.5215 (2)	1.2721 (2)	0.1249 (10)	
H16A	1.2958	−0.5157	1.2449	0.187*	
H16B	1.3372	−0.4910	1.3234	0.187*	
H16C	1.3999	−0.5980	1.3043	0.187*	
C17	1.55486 (19)	−0.4729 (2)	1.20919 (19)	0.1105 (10)	
H17A	1.5943	−0.4196	1.1506	0.166*	
H17B	1.5996	−0.5463	1.2206	0.166*	
H17C	1.5460	−0.4606	1.2708	0.166*	
C18	1.4189 (2)	−0.4036 (2)	1.0903 (2)	0.0687 (7)	
H18A	1.3454	−0.3989	1.0796	0.082*	
O16	1.05419 (13)	0.35079 (13)	0.50902 (12)	0.0826 (5)	
N10	0.99256 (16)	0.27610 (16)	0.68363 (15)	0.0612 (5)	
C34	1.0898 (2)	0.18411 (19)	0.70500 (18)	0.0913 (8)	
H34A	1.1262	0.1747	0.6415	0.137*	
H34B	1.0636	0.1181	0.7564	0.137*	
H34C	1.1450	0.1978	0.7314	0.137*	
C35	0.9053 (2)	0.2821 (2)	0.77290 (19)	0.1047 (9)	
H35A	0.8439	0.3460	0.7487	0.157*	
H35B	0.9407	0.2877	0.8212	0.157*	
H35C	0.8739	0.2164	0.8075	0.157*	
C36	0.9843 (2)	0.3510 (2)	0.5864 (2)	0.0707 (7)	
H36A	0.9193	0.4090	0.5761	0.085*	
O17	1.23621 (9)	0.22474 (10)	0.25040 (9)	0.0597 (4)	
H1A	1.2979	0.2211	0.2686	0.090*	
H1C	1.1978	0.2900	0.2365	0.090*	

### Atomic displacement parameters (Å^2^)

**Table:**

	*U*^11^	*U*^22^	*U*^33^	*U*^12^	*U*^13^	*U*^23^
O1	0.0430 (8)	0.0772 (11)	0.0370 (9)	0.0134 (8)	−0.0032 (7)	−0.0168 (9)
O2	0.0507 (8)	0.0798 (11)	0.0380 (9)	0.0137 (8)	−0.0100 (7)	−0.0156 (10)
O3	0.0440 (8)	0.0741 (11)	0.0346 (9)	0.0035 (7)	−0.0039 (7)	−0.0189 (9)
O4	0.0555 (9)	0.0804 (12)	0.0404 (10)	0.0060 (8)	0.0022 (7)	−0.0082 (9)
O5	0.0738 (10)	0.1211 (15)	0.0393 (10)	0.0138 (10)	−0.0209 (9)	0.0002 (11)
O6	0.0493 (9)	0.0871 (13)	0.0820 (13)	0.0085 (8)	−0.0174 (9)	−0.0330 (11)
O7	0.0625 (10)	0.0978 (14)	0.0652 (12)	−0.0032 (9)	0.0150 (9)	−0.0332 (12)
N1	0.0419 (10)	0.0443 (11)	0.0397 (11)	−0.0020 (8)	−0.0161 (9)	−0.0097 (10)
N2	0.0404 (9)	0.0488 (12)	0.0312 (11)	−0.0001 (8)	−0.0078 (8)	−0.0083 (10)
N3	0.0542 (12)	0.0635 (14)	0.0365 (12)	−0.0017 (10)	−0.0117 (10)	−0.0094 (11)
N4	0.0455 (12)	0.0585 (15)	0.0683 (16)	−0.0062 (10)	−0.0046 (11)	−0.0280 (14)
C1	0.0436 (12)	0.0486 (14)	0.0311 (13)	−0.0077 (10)	−0.0064 (10)	−0.0142 (12)
C2	0.0359 (11)	0.0488 (14)	0.0389 (14)	0.0008 (10)	−0.0063 (10)	−0.0188 (13)
C3	0.0422 (12)	0.0444 (14)	0.0349 (13)	0.0011 (10)	−0.0124 (10)	−0.0130 (12)
C4	0.0388 (11)	0.0487 (15)	0.0340 (13)	−0.0072 (10)	−0.0044 (10)	−0.0179 (12)
C5	0.0365 (11)	0.0480 (15)	0.0435 (14)	−0.0026 (10)	−0.0076 (11)	−0.0195 (13)
C6	0.0336 (11)	0.0401 (14)	0.0409 (14)	−0.0003 (10)	−0.0124 (10)	−0.0163 (12)
C7	0.0444 (11)	0.0678 (16)	0.0413 (14)	−0.0075 (11)	0.0025 (10)	−0.0258 (13)
C8	0.0540 (13)	0.0950 (19)	0.0485 (15)	0.0000 (13)	0.0054 (11)	−0.0334 (15)
C9	0.0430 (12)	0.0485 (14)	0.0386 (14)	−0.0045 (11)	−0.0092 (10)	−0.0167 (12)
C10	0.0392 (11)	0.0372 (13)	0.0341 (13)	−0.0046 (10)	−0.0078 (10)	−0.0128 (12)
C11	0.0430 (12)	0.0443 (14)	0.0306 (13)	−0.0080 (10)	−0.0068 (10)	−0.0115 (12)
C12	0.0400 (12)	0.0489 (15)	0.0434 (15)	−0.0004 (10)	−0.0139 (11)	−0.0173 (13)
C13	0.0366 (12)	0.0496 (15)	0.0467 (15)	−0.0030 (11)	−0.0049 (11)	−0.0216 (14)
C14	0.0527 (14)	0.0576 (16)	0.0340 (14)	−0.0092 (12)	0.0003 (11)	−0.0156 (13)
C15	0.0528 (13)	0.0512 (15)	0.0309 (13)	−0.0022 (11)	−0.0095 (10)	−0.0091 (12)
O9	0.0345 (7)	0.0843 (11)	0.0371 (9)	0.0035 (7)	−0.0047 (6)	−0.0219 (9)
O10	0.0462 (8)	0.0904 (12)	0.0355 (9)	0.0015 (8)	−0.0110 (7)	−0.0146 (10)
O11	0.0453 (8)	0.0658 (10)	0.0300 (9)	−0.0042 (7)	−0.0024 (7)	−0.0117 (8)
O12	0.0437 (8)	0.1124 (13)	0.0389 (10)	−0.0004 (9)	−0.0037 (7)	−0.0280 (10)
O13	0.0695 (9)	0.0791 (12)	0.0399 (10)	−0.0023 (9)	−0.0239 (8)	−0.0033 (9)
O14	0.0760 (11)	0.1595 (19)	0.0773 (15)	0.0073 (11)	0.0125 (10)	−0.0650 (15)
O15	0.0479 (9)	0.1076 (15)	0.0949 (15)	0.0140 (9)	−0.0156 (10)	−0.0420 (13)
N6	0.0382 (10)	0.0495 (12)	0.0339 (11)	−0.0018 (8)	−0.0139 (8)	−0.0097 (10)
N7	0.0376 (9)	0.0596 (12)	0.0304 (10)	−0.0008 (8)	−0.0094 (8)	−0.0140 (10)
N8	0.0471 (11)	0.0634 (14)	0.0379 (12)	−0.0126 (10)	−0.0128 (10)	−0.0138 (11)
N9	0.0451 (12)	0.0926 (18)	0.0819 (18)	0.0003 (11)	−0.0031 (12)	−0.0485 (17)
C19	0.0411 (11)	0.0454 (14)	0.0293 (13)	−0.0067 (10)	−0.0038 (10)	−0.0092 (11)
C20	0.0325 (11)	0.0481 (14)	0.0368 (13)	−0.0051 (10)	−0.0034 (10)	−0.0149 (12)
C21	0.0367 (11)	0.0501 (14)	0.0338 (13)	−0.0006 (10)	−0.0131 (10)	−0.0135 (12)
C22	0.0423 (12)	0.0454 (15)	0.0280 (13)	−0.0087 (10)	−0.0007 (10)	−0.0127 (13)
C23	0.0314 (10)	0.0441 (14)	0.0383 (13)	−0.0062 (10)	−0.0057 (10)	−0.0124 (12)
C24	0.0352 (11)	0.0392 (13)	0.0319 (13)	−0.0050 (10)	−0.0081 (10)	−0.0114 (12)
C25	0.0450 (12)	0.0618 (16)	0.0493 (15)	−0.0032 (11)	0.0013 (11)	−0.0233 (13)
C26	0.0482 (12)	0.0751 (17)	0.0421 (14)	−0.0094 (11)	0.0052 (10)	−0.0251 (13)
C27	0.0417 (11)	0.0457 (14)	0.0340 (13)	−0.0074 (10)	−0.0061 (10)	−0.0136 (12)
C28	0.0355 (11)	0.0485 (14)	0.0321 (13)	−0.0095 (10)	−0.0084 (9)	−0.0139 (12)
C29	0.0383 (11)	0.0485 (14)	0.0294 (13)	−0.0103 (10)	−0.0048 (10)	−0.0150 (12)
C30	0.0399 (12)	0.0474 (15)	0.0498 (15)	0.0004 (10)	−0.0163 (11)	−0.0168 (14)
C31	0.0344 (12)	0.0663 (17)	0.0533 (16)	−0.0037 (11)	−0.0024 (11)	−0.0315 (15)
C32	0.0510 (13)	0.0859 (18)	0.0380 (15)	−0.0095 (13)	−0.0034 (11)	−0.0286 (15)
C33	0.0426 (12)	0.0713 (16)	0.0314 (14)	−0.0082 (11)	−0.0075 (10)	−0.0169 (13)
O8	0.0799 (12)	0.1070 (15)	0.0471 (12)	−0.0114 (10)	−0.0173 (9)	−0.0147 (12)
N5	0.0610 (12)	0.0592 (13)	0.0424 (13)	−0.0091 (10)	−0.0074 (10)	−0.0115 (11)
C16	0.119 (2)	0.104 (2)	0.099 (2)	−0.047 (2)	0.0239 (19)	−0.007 (2)
C17	0.0744 (17)	0.162 (3)	0.0693 (19)	0.0021 (17)	−0.0319 (15)	−0.033 (2)
C18	0.0560 (16)	0.083 (2)	0.071 (2)	−0.0053 (14)	−0.0170 (15)	−0.0374 (19)
O16	0.0766 (11)	0.0999 (14)	0.0364 (10)	0.0050 (10)	−0.0109 (8)	−0.0131 (10)
N10	0.0809 (13)	0.0586 (14)	0.0384 (13)	−0.0199 (11)	0.0006 (11)	−0.0173 (12)
C34	0.1037 (19)	0.085 (2)	0.0574 (18)	0.0015 (17)	−0.0343 (15)	−0.0085 (16)
C35	0.131 (2)	0.095 (2)	0.074 (2)	−0.0475 (18)	0.0401 (18)	−0.0392 (19)
C36	0.0670 (16)	0.0641 (19)	0.062 (2)	−0.0025 (14)	−0.0107 (15)	−0.0177 (18)
O17	0.0451 (7)	0.0630 (10)	0.0647 (10)	0.0003 (7)	−0.0195 (7)	−0.0226 (9)

### Geometric parameters (Å, °)

**Table:**

O1—C2	1.3641 (19)	N7—C28	1.3440 (19)
O1—C7	1.4270 (19)	N7—H7	0.8600
O2—C3	1.357 (2)	N8—C29	1.445 (2)
O2—H2	0.8200	N9—C31	1.459 (2)
O3—C4	1.369 (2)	C19—C20	1.383 (2)
O3—C8	1.4220 (18)	C19—C24	1.383 (2)
O4—N3	1.2217 (17)	C19—H19A	0.9300
O5—N3	1.2153 (18)	C20—C21	1.386 (2)
O6—N4	1.2279 (19)	C21—C22	1.397 (2)
O7—N4	1.232 (2)	C22—C23	1.373 (2)
N1—C9	1.2714 (19)	C23—C24	1.398 (2)
N1—N2	1.3813 (18)	C23—H23A	0.9300
N2—C10	1.3450 (19)	C24—C27	1.455 (2)
N2—H2A	0.8600	C25—H25A	0.9600
N3—C11	1.451 (2)	C25—H25B	0.9600
N4—C13	1.453 (2)	C25—H25C	0.9600
C1—C2	1.380 (2)	C26—H26A	0.9600
C1—C6	1.388 (2)	C26—H26B	0.9600
C1—H1B	0.9300	C26—H26C	0.9600
C2—C3	1.398 (2)	C27—H27A	0.9300
C3—C4	1.397 (2)	C28—C29	1.415 (2)
C4—C5	1.372 (2)	C28—C33	1.420 (2)
C5—C6	1.392 (2)	C29—C30	1.381 (2)
C5—H5A	0.9300	C30—C31	1.358 (2)
C6—C9	1.452 (2)	C30—H30A	0.9300
C7—H7B	0.9600	C31—C32	1.386 (3)
C7—H7C	0.9600	C32—C33	1.352 (2)
C7—H7D	0.9600	C32—H32A	0.9300
C8—H8A	0.9600	C33—H33A	0.9300
C8—H8B	0.9600	O8—C18	1.193 (2)
C8—H8C	0.9600	N5—C18	1.324 (3)
C9—H9A	0.9300	N5—C17	1.434 (2)
C10—C11	1.412 (2)	N5—C16	1.441 (3)
C10—C15	1.418 (2)	C16—H16A	0.9600
C11—C12	1.379 (2)	C16—H16B	0.9600
C12—C13	1.354 (2)	C16—H16C	0.9600
C12—H12A	0.9300	C17—H17A	0.9600
C13—C14	1.389 (2)	C17—H17B	0.9600
C14—C15	1.357 (2)	C17—H17C	0.9600
C14—H14A	0.9300	C18—H18A	0.9300
C15—H15A	0.9300	O16—C36	1.216 (2)
O9—C20	1.3654 (18)	N10—C36	1.320 (3)
O9—C25	1.4290 (18)	N10—C34	1.431 (2)
O10—C21	1.3567 (19)	N10—C35	1.459 (2)
O10—H10	0.8200	C34—H34A	0.9600
O11—C22	1.3674 (19)	C34—H34B	0.9600
O11—C26	1.4262 (18)	C34—H34C	0.9600
O12—N8	1.2236 (17)	C35—H35A	0.9600
O13—N8	1.2213 (17)	C35—H35B	0.9600
O14—N9	1.223 (2)	C35—H35C	0.9600
O15—N9	1.228 (2)	C36—H36A	0.9300
N6—C27	1.2743 (18)	O17—H1A	0.8434
N6—N7	1.3753 (17)	O17—H1C	0.8411
			
C2—O1—C7	118.08 (15)	O10—C21—C20	117.24 (16)
C3—O2—H2	109.5	O10—C21—C22	122.92 (17)
C4—O3—C8	117.50 (14)	C20—C21—C22	119.83 (17)
C9—N1—N2	116.51 (15)	O11—C22—C23	125.91 (17)
C10—N2—N1	117.98 (15)	O11—C22—C21	114.44 (17)
C10—N2—H2A	121.0	C23—C22—C21	119.65 (18)
N1—N2—H2A	121.0	C22—C23—C24	120.34 (17)
O5—N3—O4	121.83 (19)	C22—C23—H23A	119.8
O5—N3—C11	118.69 (18)	C24—C23—H23A	119.8
O4—N3—C11	119.45 (17)	C19—C24—C23	120.03 (17)
O6—N4—O7	123.38 (18)	C19—C24—C27	119.58 (17)
O6—N4—C13	118.2 (2)	C23—C24—C27	120.36 (16)
O7—N4—C13	118.4 (2)	O9—C25—H25A	109.5
C2—C1—C6	119.96 (17)	O9—C25—H25B	109.5
C2—C1—H1B	120.0	H25A—C25—H25B	109.5
C6—C1—H1B	120.0	O9—C25—H25C	109.5
O1—C2—C1	125.71 (17)	H25A—C25—H25C	109.5
O1—C2—C3	114.22 (17)	H25B—C25—H25C	109.5
C1—C2—C3	120.06 (17)	O11—C26—H26A	109.5
O2—C3—C4	123.09 (18)	O11—C26—H26B	109.5
O2—C3—C2	117.15 (16)	H26A—C26—H26B	109.5
C4—C3—C2	119.76 (18)	O11—C26—H26C	109.5
O3—C4—C5	126.32 (17)	H26A—C26—H26C	109.5
O3—C4—C3	113.97 (17)	H26B—C26—H26C	109.5
C5—C4—C3	119.71 (18)	N6—C27—C24	120.03 (17)
C4—C5—C6	120.61 (17)	N6—C27—H27A	120.0
C4—C5—H5A	119.7	C24—C27—H27A	120.0
C6—C5—H5A	119.7	N7—C28—C29	124.55 (17)
C1—C6—C5	119.89 (18)	N7—C28—C33	119.76 (17)
C1—C6—C9	118.85 (18)	C29—C28—C33	115.69 (16)
C5—C6—C9	121.21 (17)	C30—C29—C28	121.36 (17)
O1—C7—H7B	109.5	C30—C29—N8	116.69 (18)
O1—C7—H7C	109.5	C28—C29—N8	121.89 (16)
H7B—C7—H7C	109.5	C31—C30—C29	120.20 (19)
O1—C7—H7D	109.5	C31—C30—H30A	119.9
H7B—C7—H7D	109.5	C29—C30—H30A	119.9
H7C—C7—H7D	109.5	C30—C31—C32	120.49 (18)
O3—C8—H8A	109.5	C30—C31—N9	120.3 (2)
O3—C8—H8B	109.5	C32—C31—N9	119.2 (2)
H8A—C8—H8B	109.5	C33—C32—C31	119.96 (19)
O3—C8—H8C	109.5	C33—C32—H32A	120.0
H8A—C8—H8C	109.5	C31—C32—H32A	120.0
H8B—C8—H8C	109.5	C32—C33—C28	122.20 (19)
N1—C9—C6	120.06 (18)	C32—C33—H33A	118.9
N1—C9—H9A	120.0	C28—C33—H33A	118.9
C6—C9—H9A	120.0	C18—N5—C17	119.7 (2)
N2—C10—C11	124.94 (17)	C18—N5—C16	122.0 (2)
N2—C10—C15	119.70 (18)	C17—N5—C16	118.0 (2)
C11—C10—C15	115.35 (17)	N5—C16—H16A	109.5
C12—C11—C10	121.97 (18)	N5—C16—H16B	109.5
C12—C11—N3	116.52 (18)	H16A—C16—H16B	109.5
C10—C11—N3	121.43 (17)	N5—C16—H16C	109.5
C13—C12—C11	119.99 (19)	H16A—C16—H16C	109.5
C13—C12—H12A	120.0	H16B—C16—H16C	109.5
C11—C12—H12A	120.0	N5—C17—H17A	109.5
C12—C13—C14	120.62 (18)	N5—C17—H17B	109.5
C12—C13—N4	120.3 (2)	H17A—C17—H17B	109.5
C14—C13—N4	119.1 (2)	N5—C17—H17C	109.5
C15—C14—C13	119.77 (18)	H17A—C17—H17C	109.5
C15—C14—H14A	120.1	H17B—C17—H17C	109.5
C13—C14—H14A	120.1	O8—C18—N5	126.1 (2)
C14—C15—C10	122.29 (19)	O8—C18—H18A	116.9
C14—C15—H15A	118.9	N5—C18—H18A	116.9
C10—C15—H15A	118.9	C36—N10—C34	120.1 (2)
C20—O9—C25	117.99 (13)	C36—N10—C35	122.7 (2)
C21—O10—H10	109.5	C34—N10—C35	117.2 (2)
C22—O11—C26	117.57 (14)	N10—C34—H34A	109.5
C27—N6—N7	116.61 (15)	N10—C34—H34B	109.5
C28—N7—N6	117.08 (15)	H34A—C34—H34B	109.5
C28—N7—H7	121.5	N10—C34—H34C	109.5
N6—N7—H7	121.5	H34A—C34—H34C	109.5
O13—N8—O12	122.04 (17)	H34B—C34—H34C	109.5
O13—N8—C29	118.88 (17)	N10—C35—H35A	109.5
O12—N8—C29	119.07 (17)	N10—C35—H35B	109.5
O14—N9—O15	123.7 (2)	H35A—C35—H35B	109.5
O14—N9—C31	118.4 (2)	N10—C35—H35C	109.5
O15—N9—C31	117.8 (2)	H35A—C35—H35C	109.5
C20—C19—C24	119.60 (17)	H35B—C35—H35C	109.5
C20—C19—H19A	120.2	O16—C36—N10	125.8 (2)
C24—C19—H19A	120.2	O16—C36—H36A	117.1
O9—C20—C19	124.59 (17)	N10—C36—H36A	117.1
O9—C20—C21	114.87 (17)	H1A—O17—H1C	105.8
C19—C20—C21	120.52 (17)		
			
C9—N1—N2—C10	−179.87 (16)	C25—O9—C20—C21	159.79 (17)
C7—O1—C2—C1	7.0 (3)	C24—C19—C20—O9	−176.94 (17)
C7—O1—C2—C3	−173.36 (15)	C24—C19—C20—C21	1.5 (3)
C6—C1—C2—O1	179.75 (17)	O9—C20—C21—O10	−0.4 (2)
C6—C1—C2—C3	0.1 (3)	C19—C20—C21—O10	−178.96 (16)
O1—C2—C3—O2	−0.3 (2)	O9—C20—C21—C22	178.79 (16)
C1—C2—C3—O2	179.40 (16)	C19—C20—C21—C22	0.2 (3)
O1—C2—C3—C4	179.75 (16)	C26—O11—C22—C23	−1.1 (2)
C1—C2—C3—C4	−0.6 (3)	C26—O11—C22—C21	178.48 (15)
C8—O3—C4—C5	1.2 (3)	O10—C21—C22—O11	−2.1 (3)
C8—O3—C4—C3	−179.48 (15)	C20—C21—C22—O11	178.75 (16)
O2—C3—C4—O3	1.0 (3)	O10—C21—C22—C23	177.47 (17)
C2—C3—C4—O3	−179.05 (16)	C20—C21—C22—C23	−1.7 (3)
O2—C3—C4—C5	−179.70 (17)	O11—C22—C23—C24	−179.07 (17)
C2—C3—C4—C5	0.3 (3)	C21—C22—C23—C24	1.4 (3)
O3—C4—C5—C6	179.72 (17)	C20—C19—C24—C23	−1.8 (3)
C3—C4—C5—C6	0.5 (3)	C20—C19—C24—C27	176.25 (16)
C2—C1—C6—C5	0.6 (3)	C22—C23—C24—C19	0.3 (3)
C2—C1—C6—C9	−176.73 (16)	C22—C23—C24—C27	−177.67 (15)
C4—C5—C6—C1	−0.9 (3)	N7—N6—C27—C24	178.82 (14)
C4—C5—C6—C9	176.36 (16)	C19—C24—C27—N6	−175.15 (18)
N2—N1—C9—C6	−179.34 (14)	C23—C24—C27—N6	2.8 (2)
C1—C6—C9—N1	173.56 (17)	N6—N7—C28—C29	178.06 (15)
C5—C6—C9—N1	−3.8 (3)	N6—N7—C28—C33	−2.0 (2)
N1—N2—C10—C11	176.00 (15)	N7—C28—C29—C30	−177.35 (18)
N1—N2—C10—C15	−3.2 (2)	C33—C28—C29—C30	2.7 (2)
N2—C10—C11—C12	−179.73 (17)	N7—C28—C29—N8	5.5 (3)
C15—C10—C11—C12	−0.5 (2)	C33—C28—C29—N8	−174.43 (16)
N2—C10—C11—N3	−3.2 (3)	O13—N8—C29—C30	6.2 (2)
C15—C10—C11—N3	176.03 (16)	O12—N8—C29—C30	−172.52 (16)
O5—N3—C11—C12	−2.0 (3)	O13—N8—C29—C28	−176.60 (16)
O4—N3—C11—C12	176.30 (17)	O12—N8—C29—C28	4.7 (3)
O5—N3—C11—C10	−178.76 (18)	C28—C29—C30—C31	−0.6 (3)
O4—N3—C11—C10	−0.4 (3)	N8—C29—C30—C31	176.67 (17)
C10—C11—C12—C13	0.6 (3)	C29—C30—C31—C32	−2.1 (3)
N3—C11—C12—C13	−176.08 (16)	C29—C30—C31—N9	178.97 (17)
C11—C12—C13—C14	−0.2 (3)	O14—N9—C31—C30	−174.3 (2)
C11—C12—C13—N4	178.68 (16)	O15—N9—C31—C30	4.8 (3)
O6—N4—C13—C12	7.0 (3)	O14—N9—C31—C32	6.8 (3)
O7—N4—C13—C12	−171.76 (19)	O15—N9—C31—C32	−174.12 (19)
O6—N4—C13—C14	−174.10 (18)	C30—C31—C32—C33	2.6 (3)
O7—N4—C13—C14	7.2 (3)	N9—C31—C32—C33	−178.49 (19)
C12—C13—C14—C15	−0.2 (3)	C31—C32—C33—C28	−0.3 (3)
N4—C13—C14—C15	−179.17 (18)	N7—C28—C33—C32	177.80 (18)
C13—C14—C15—C10	0.3 (3)	C29—C28—C33—C32	−2.2 (3)
N2—C10—C15—C14	179.29 (17)	C17—N5—C18—O8	−0.9 (4)
C11—C10—C15—C14	0.0 (3)	C16—N5—C18—O8	−175.0 (2)
C27—N6—N7—C28	−179.99 (17)	C34—N10—C36—O16	−0.5 (3)
C25—O9—C20—C19	−21.7 (2)	C35—N10—C36—O16	179.7 (2)

### Hydrogen-bond geometry (Å, °)

**Table:**

*D*—H···*A*	*D*—H	H···*A*	*D*···*A*	*D*—H···*A*
O2—H2···O16	0.82	1.89	2.610 (2)	145
N2—H2A···O4	0.86	2.05	2.637 (2)	125
N2—H2A···O17^i^	0.86	2.40	3.1141 (19)	140
O10—H10···O8	0.82	1.89	2.604 (2)	145
O10—H10···O11	0.82	2.27	2.7030 (17)	113
N7—H7···O12	0.86	2.06	2.642 (2)	125
N7—H7···O17	0.86	2.39	3.118 (2)	143
O17—H1A···O9^ii^	0.84	2.22	2.9936 (16)	153
O17—H1A···O10^ii^	0.84	2.42	3.1042 (17)	139
O17—H1C···O1	0.84	2.34	3.0790 (19)	147
O17—H1C···O2	0.84	2.44	3.1811 (18)	147

Symmetry codes: (i) −*x*+2, −*y*+1, −*z*; (ii) −*x*+3, −*y*, −*z*+1.
